# Interdisciplinary design education: development of an elective course in architecture and engineering departments

**DOI:** 10.1186/s44147-021-00010-2

**Published:** 2021-09-22

**Authors:** A. M. Badawi, M. R. Abdullah

**Affiliations:** Faculty of Engineering, Cairo University, Giza, Egypt

**Keywords:** Architectural education, Design studio, Interdisciplinary design, Architecture, Engineering, and Construction (AEC)

## Abstract

Collaboration among disciplines is becoming a standard practice in Architecture, Engineering, and Construction (AEC) industry. However, limited studies have addressed the involvement of interdisciplinarity into architectural undergraduate curricula. The study seeks to expand the literature on this topic, namely by offering an alternative model for teaching an Interdisciplinary Design Course (IDC), mainly in architectural engineering departments, with the participation of engineering departments. The authors hypothesize that by the adoption of the IDC, architecture students would have a better understanding of the nature of AEC interdisciplinary design knowledge. The study aims to highlight the value of the IDC and to test the hypothesis. A qualitative research methodology has been adopted, including the design of an experiment and then the application of a case study comprising four instructors and 24 students from four departments in the Faculty of Engineering, Cairo University. Students’ design process, teamwork attitude, and own experiences on the IDC have been recorded using direct observation, interviews, and surveys. Data have been analyzed using descriptive statistics to identify the effectiveness and challenges of the experiment, in addition to the comparison with the traditional design studio. The results have shown the students’ satisfaction with collaboration with their peers from other disciplines, as it boosted their understanding of the integrated design process and increased their knowledge about each other’s discipline. In addition, architecture students commended the IDC much more than the traditional design studio.

**Supplementary Information:**

The online version contains supplementary material available at 10.1186/s44147-021-00010-2.

## Introduction

The real practice of Architecture, Engineering, and Construction (AEC) is based on collaboration among disciplines [1]. The design of buildings has a level of complexity that makes it unexpectable for the architect alone to integrate all building design aspects [2]. In addition, the implementation of AEC’s projects, which are usually complicated and large in scale, requires interaction among stakeholders from different disciplines: architects, clients, users, and engineers [3]. Hence, the AEC industry continues to seek graduates that possess interdisciplinary collaboration and communication skills [3]. Nevertheless, a great effort is still needed to address the needs and challenges of the current interdisciplinary education [1]. A recent study that targeted 100 fresh graduates and senior students was conducted aiming to find what are today’s needed competencies for architecture students to be employed in the Egyptian market. Khodeir and Nessim concluded that the current architectural education rarely helps students to acquire the skills related to teamwork, such as active listening, communication, leadership, and conflict resolution [4]. Accordingly, filling such education-to-practice gap requires graduation of well-qualified students equipped with teamwork and interdisciplinary skills [5].

The term “Interdisciplinary” is an adjective that describes the interaction that occurs among two or more disciplines. Such interaction is broader than “multi-disciplinary” teamwork or collaboration, which are considered as one-discipline-based terms [6]. According to Borrego and Cutler, multi-disciplinarity is less integrative than interdisciplinarity. The latter is more suitable for engineering projects, as it requires high integration among the involved members from different disciplines to develop a synthesized product [7]. Although it has been found that interdisciplinary works are common in engineering practice and education, they however are almost rarely adopted in current architectural schools [8].

In essence, building an interdisciplinary course combining architecture and engineering is a challenge due to the pedagogical differences between the two pillars: architecture and engineering [9]. To overcome such challenge, many educators call for the adoption of Problem-Based Learning approach as a teaching strategy [10]. However, the Project-Based Learning approach, carried out in the design studio, has been introduced to be more valuable in engineering programs [11, 12]. They are also known as Capstone Courses, which are prepared to integrate students’ previous coursework into a comprehensive, team-based course [13]. Above all, collaboration among educators from architecture and other involved disciplines is the key factor for the successful construction of such courses [9].

Various attempts at interdisciplinary approaches in design education were made since the late 1980s [14]. Two significant papers reviewed a number of studies that addressed such attempts [1, 14]. Firstly, Irizarry et al. analyzed various academic institutions that adopted the AEC collaborative and interdisciplinary pedagogical models from 1995 to 2009. They found that such models have yielded positive results. Nevertheless, they argued that these contributions were not sufficient to comprehensively study interdisciplinarity in education since they had limited tests and applications [1]. Secondly, Kalyanaraman et al. reviewed another list of studies relevant to the interdisciplinary curriculum in AEC. They referred to the growth of attention in AEC interdisciplinary education from the 1990s, as their timeline shows that studies before 1990s addressed interdisciplinarity in general, while those after the 1990s were specialized in the AEC sector [14].

However, limited attempts have addressed the pedagogical challenges to undergraduate interdisciplinary courses [9]. A part of such studies has addressed theoretical basics while the other part has recorded application attempts in design studios. Firstly, Smith has introduced an early attempt at addressing an AEC capstone course, ending up describing the best practices of creation of interdisciplinary teams, projects, assignments, students’ critique, and grading [13]. Grading students in such courses has been considered a challenge. This is due to not only the subjectivity manner in the architectural design studio, but also the interdisciplinarity itself. Team-based assessment is a complicated issue that is hard to resolve, because each student’s grade is based on his/her team performance [15]. Ghonim has offered a framework for interdisciplinary graduation projects, showing that each student from a particular specialization has his/her own Intended Learning Outcomes (ILOs) and can be easily evaluated, while all students are still able to engage in and solve real-life problems [8]. Solonsky and Parfitt have published a significant paper that provides an expanding theoretical basis of how to construct an AEC capstone program [16]. They have described trends and successes of 9 years of experience in offering such programs. They have also summarized the best practices for future implementation. Such practices however need to be tested by other programs and courses.

A recent study has adopted an experimental course of an AEC interdisciplinary design [17]. After selecting a pilot project with a limited scope of design, Ali has designed a case study of three different programs: the architecture, building construction, and construction management and engineering. The students were requested to design building systems including mechanical and lighting-electrical systems, which however was not at the core of their specialties. It could be more beneficial to involve students from mechanical and electrical departments, who had the technical expertise that is not covered in architecture and construction curricula.

The present study attempts to further expand the literature on interdisciplinary design education, offering an alternative model for collaborative teaching, mainly in the architecture department, and with the involvement of related engineering departments, i.e., structural, electrical, and mechanical engineering departments. The authors hypothesize that architecture students working on an interdisciplinary project would understand the nature of AEC interdisciplinary design knowledge, better than if working on a traditional project-based design studio.

The objective of this paper is to highlight the value of interdisciplinary design studio employed as an elective course in the architecture department, in addition to test the hypothesis, through a designed case study of instructors and senior students from different departments. It is worth mentioning that the meant interdisciplinary design course is that by which students from different disciplines participate in the design, get critiqued, and graded by an interdisciplinary team of academicians and experts. It is different from the traditional mono-discipline courses that provide a background about other disciplines’ knowledge and that are restricted to only architecture instructors and students.

## Methods

The study adopts a qualitative research methodology. To start with, the study has synthesized a pedagogical framework for constructing an Interdisciplinary Design Course (IDC), based on the literature, and aiming to find the best practices of teaching IDCs. Based on the framework, an experiment has been designed and then implemented with a case study of 24 fourth-year students from four different departments. Students’ design processes and teamwork attitudes have been recorded using direct observation and interviews during the experiment. In addition, students’ own experiences have been registered using a post-experiment survey, which has been sent to the students after the last day of the experiment. Data have been analyzed using descriptive statistics, directly to identify the effectiveness and encountered challenges of the experiment. Results have been compared to the Traditional Design Studio (TDS) to test the hypothesis.

## Framework

The study has selected five papers that documented experiments of teaching interdisciplinary design, aiming to figure out the best practices for constructing such experiments, e.g., course durations, learning objectives, and project selection criteria. Table 1 presents the chosen programs and their specifications. In choosing the programs, priority was given to those with the maximum number of years of experience applying such courses. Other criteria were related to the abundance of information available in the paper, as well as the clarity of the course outline. Details of the framework are presented.

**Table 1:** The chosen programs that documented experiments of teaching interdisciplinary design

Paper	University	Years of experience	Undergraduate/graduate	Course duration
[17]	Texas A&M University	-	UG & Gr.	1 semester
[16]	The Pennsylvania State University	9 years	UG & Gr.	1–2 semesters
[18]		5 years		
[19]	The Pennsylvania State University	10 years	UG (extra) & Gr.	1 semester
[15]	California Polytechnic State University	60 years (collaboration), 3 years (the course)	UG	1 quarter (7–10 weeks)

### Course objectives and assessment criteria

Each program should create a set of learning objectives as to represent its own ideologies and circumstances, yet meeting the criteria of an accreditation organization [18]. Guthrie et al. have categorized the objectives into two main parts, based on the type of intended developed skill (product-focused skills and teamwork skills), namely, (1) creating an interdisciplinary design and (2) functioning effectively on interdisciplinary teams [15]. The study has proposed the following assessment criteria and their respective ILOs based on the analysis of the aforementioned selected studies, shown in Table 2.

**Table 2:** Assessment criteria and respective ILOs

Assessment criteria		Respective ILOs
**Students’ projects**	• Discipline technical content (drawings, reports) with the consideration of the design principles	1. Increase knowledge of discipline depth. 2. Utilize previous knowledge of coursework. 3. Expansion of breadth and depth knowledge by self-learning. 4. Develop proper work processes/paths. 5. Obtain more powerful skillsets revolving around modeling tools. 6. Expand the ability to research, evaluate, and implement new technology.
	• Presentation skills and clarity of work	
	• Technical integration content (presentation, drawings, reports)	7. Gain a better understanding of the integrated process and the give and how real projects are developed and designed.
**Teamwork skills**	• Productivity	8. Develop a team approach that takes precedence over individual disciplines.
	• Effort management	
	• Communication effectiveness	
	• Whole performance of the team members	

### Course duration

**Fig. 1:**
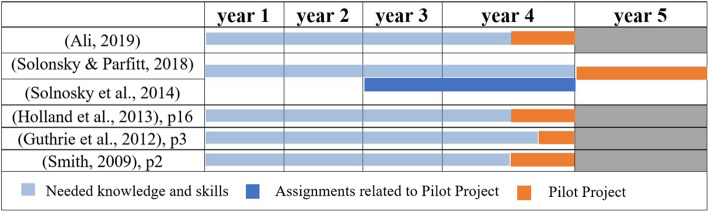
The timelines of the programs of the selected studies

Usually, interdisciplinary courses are being run at the latest academic year or senior level, which is the culmination of architectural education [17]. Moreover, Ghonim et al. call for adopting interdisciplinary design as a part of the graduation project, which represents this culmination [8]. However, only one semester may not be sufficient for students to fully attain needed competencies for applying the pilot project, rather, students need 1 year at least [16, 17]. Therefore, in some programs, technical engineering and management skills are provided to students from the very beginning of enrollment (3–4 years of classes) [16]. Accordingly, the nature of the last semester/year of such programs (pilot project) is not to teach new topics but to let students synthesize and apply previously learned skills to a real project [18]. Figure 1 presents the timelines of the programs of the aforementioned selected studies, showing the pilot project’s duration in each program, as well as the duration of the needed knowledge and skills provided in the previous years.

## Experimenting interdisciplinary design

### Design of the case study

The experiment of the interdisciplinary design was carried out in the academic year 2019–2020 as an elective course, for 24 fourth-year students from four different departments in the Faculty of Engineering, Cairo University, namely (1) Architectural Engineering (ARCH), (2) Structural Engineering (STR), (3) Electrical Power Engineering (ELEC), and (4) Mechanical Power Engineering (MECH). Coordination with the last three departments started 2 months before the semester’s beginning. Instructors were informed by the authors of the experiment’s idea. They were then requested to assign at least four volunteer students from their departments to join the experiment. Instructors were requested also to participate in supervision. At that time, the faculty was not applying interdisciplinary courses or programs, which made a considerable challenge in the preparation of the experiment, since neither instructors nor institution was ready with proper experiences and facilities.

However, the selection procedures of ARCH students were easier. After publishing a video to architecture students explaining the idea and encouraging them to volunteer, an online survey was conducted, targeting those who were interested in joining the course. The survey contained questions regarding the academic performance level, the desire extent to join the course, and the level of mastering Revit software. Out of 29 respondents from 140 architecture students studying in the 4th year, 12 architecture students were chosen, taking into consideration (1) the highest desire to join the course, the highest level of mastering Revit software, and the variety of the academic performance level. The chosen students were then divided into four teams, three students in each. By the end of the introduction lecture at the first week, all students were requested to get to know each other, so the architecture teams can choose their partners from the other disciplines (each team has 3 ARCH, 1 STR, 1 MECH, and 1 ELEC). The experiment was run as a competition among the four teams. The students were to meet on campus 2 h a week, according to the proposed schedule.

### Selection of the pilot project

In the semester prior to the experiment, ARCH students had been through a traditional project-based studio that provided a background about other disciplines’ knowledge. The pilot project’s specifications were designed to be close to that of the project assigned in the previous semester, including design duration and project size and typology, so the comparison between traditional and the IDC could be more reliable. In addition, the specifications were designed in which to ensure equal challenges among students from different disciplines. The design challenges and their respective disciplines are presented in Table 3.

**Table 3:** Design challenges and respective disciplines

Design challenge	Discipline			
	ARCH	Civil	MECH	ELEC
Coexistence with surroundings	●			
Contemporary and smart building technologies	●			●
Sustainability and future adaptability	●	●	●	●
Safety	●	●	●	●
Energy efficiency			●	●

Although the chosen project was not supported by a real client, specifications were based on real circumstances. The pilot project was a cinema complex intended to be built as a part of a larger complex compound (commercial, offices, recreational). The project land was owned by one of the investment companies, in a prestigious setting on 26 of July route, in El-Sheikh Zayed City. The ILOs and assessment criteria of the project were set as what previously mentioned in Table 2. The project specifications and required deliverables were distributed to the students at the first week. The students should use a software of Building Information Modeling (BIM) technology. BIM is a recent management platform aligning with the recent shift in the AEC industries that is from fragmented deliverables to a single database management [17].

### Teamwork challenges

During the experiment, semi-structured interviews were conducted with a teammate in each team. The questions were regarding productivity, interaction among members, and communication efficiency, which collectively indicated the level of teamwork efficiency. It could be concluded from the discussions that ARCH students were much more interactive with each other, while less interactive with the other disciplines. In addition, all teams expressed the dominant role of ARCH at the first stages of design, one team even stated that the role of the other disciplines was almost ignored at the schematic design phase. Meeting manners were also different among the teams. Two teams were conducting meetings with the participation of all disciplines, which was expected. They claimed that productivity gets higher in the multi-disciplinary meeting rather than while working separately. However, the ARCH members of another team were working either solo or in pairs with each discipline member when needed. The members of the fourth team were not working in parallel, they were rather exchanging the same working Revit file and developing it consecutively. Communication modalities were almost the same for all teams, which were direct conversations and personal laptops. Other than physical meetings on campus, the students contacted online via WhatsApp as extra meeting hours spent working on the project.

### Design products

**Fig. 2:**
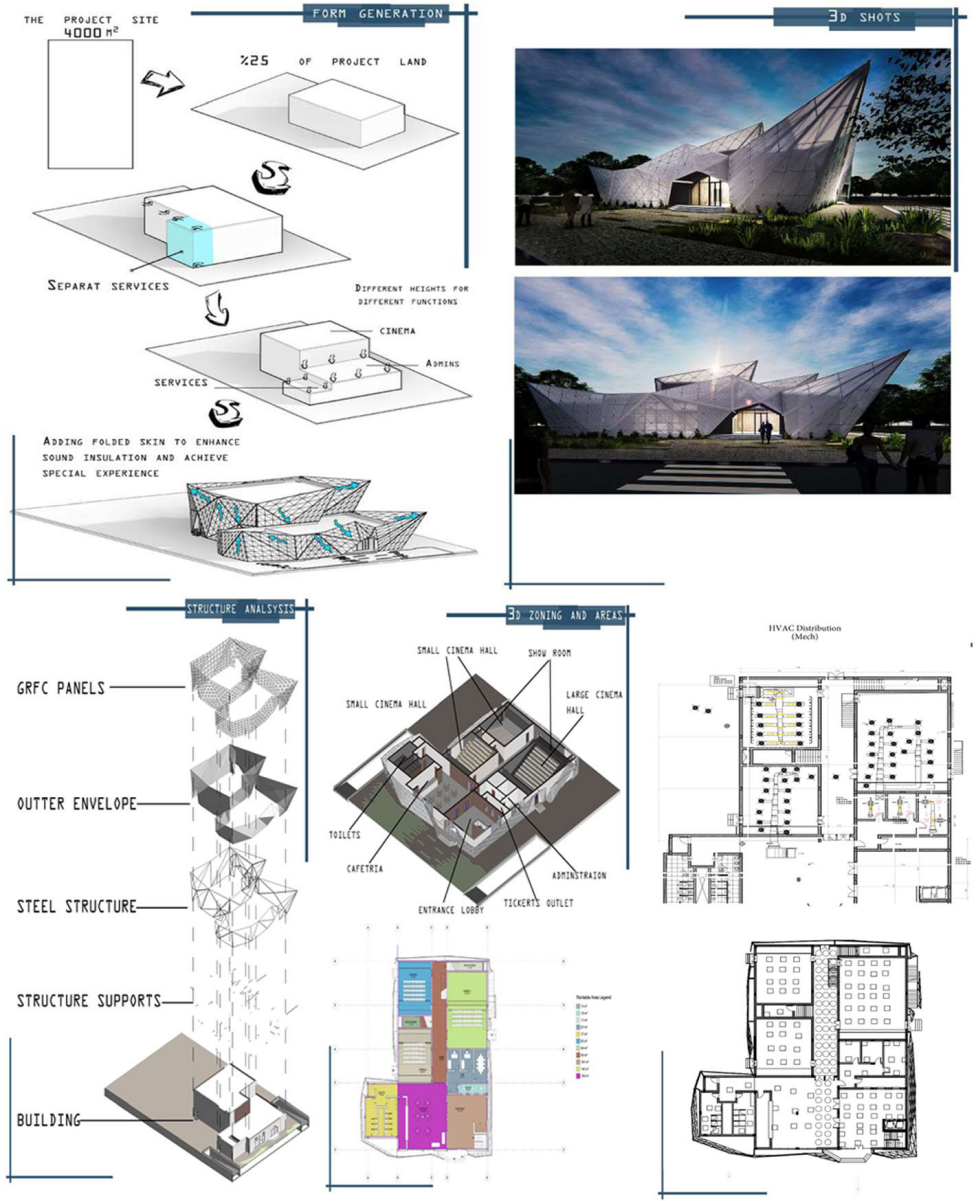
A part of the submitted report of team 1

At the last weeks, students prepared the presentation of their projects, beginning with clarifying the project’s concept, functional zoning, and form generation, followed by technical drawings of all disciplines. The detailed tasks and output format are illustrated in Table 4. Figure 2 shows a part of a team’s submitted report.

**Table 4:** Specific tasks and output format

ARCH	STR	MECH	ELEC	Task		Output format	
●				Architectural concept and visual studies		Diagrams, sketches, 3D shots	A4 + Pres.
●				Architectural drawings		Plans, sections, elevations, and 3Ds	A3
	●			Structural analysis		Structural plans	A3
						Report	A4
●	●			Site logistics	Provision and discussion of construction site logistics plan	Report	A4 + Pres.
●			●	Environmental systems	Discussion of renewable energy facilities to be considered	Report	A4 + Pres.
		●		HVAC	Provision of the required HVAC systems	HVAC dist. layout (with legends)	A3
			●	Electrical power	Provision of the required electrical + lighting dist.	Electrical and lighting dist. layout (with legends)	A3
●	●	●	●	Value analysis and cost estimation		Bill of quantities	A4
●	●	●	●	Coordination and clash detection	Solving the detected clashes and provision of a coordinated design	Coordinated ceiling plan	A3
						BIM model of coordinated building	DVD

### Post-experiment survey

After the experiment was finished, an online questionnaire was sent to the students from the four disciplines (see the Additional file 1). The questionnaire was divided into three parts. The first part (8 questions) was regarding students’ understanding of integrated design aspects and awareness of each other’s discipline. The questions of the second part (3 questions) were open-ended questions regarding the advantages and the encountered challenges of the experience and the suggestions for it. The third part was assigned only to ARCH students, asking about how much the experience of interdisciplinarity had enhanced their design capabilities compared to the TDS. The survey consisted of 14 questions. Eleven questions were numeric, using a 5-point Likert scale. The other three questions were open-ended, analyzed using descriptive statistics. The number of valid responses from students was 22, out of 24 students enrolled in the course from the four departments (response rate 92%). The respondents’ profiles are shown in Table 5.

**Table 5:** The respondents’ profiles

Department	No. of students	No. of valid responses	% of responses
Architectural Engineering	12	12	100
Structural Engineering	4	4	100
Electrical Power Engineering	4	3	75
Mechanical Power Engineering	4	3	75
Total	**24**	**22**	**92**

## Results and discussion

Students were asked about the understanding of different design aspects considering the knowledge acquired from other disciplines. The rating was calculated by giving 5 points if the experiment helped him/her to excellently understand the design aspect. On the contrary, 1 point was given if the experiment did not help him/her at all. Table 6 shows the averages of students’ ratings for the aspects. The ratings ranged between 3.08 and 4.00. In addition, the results show that the architectural aspects have the highest ratings among all disciplines, which represents the ability of ARCH students to better deliver their ideas.

**Table 6:** The averages of students’ ratings

	ARCH	SRT	ELEC	MECH	AVG.
Form and esthetics	4.08	4	3	3.67	**3.69**
Architectural functions	4.67	4	3.33	4	**4**
Delivery presentation	4	3.5	4.33	4	**3.96**
Structural systems	4.67	4.75	2.67	3.33	**3.86**
Electrical systems	3.08	2.25	3.33	3.67	**3.08**
Mechanical systems	4	2.5	3.67	5	**3.79**

All the students indicated that working, communication or collaboration with the students from the other disciplines, was the main advantage of the experience. Particularly, four students (18%) claimed that they had a clear picture of the nature of other disciplines’ tasks. However, four ARCH students (18%) complained about the lack of communication with the other disciplines, attributing the reason to the other disciplines’ unawareness of architectural work. ARCH students described their experiences regarding working with other disciplines as in Table 7.

**Table 7:** The students’ comments regarding working with other disciplines

**Student 1**	*I could understand the way of thinking of the other disciplines that is beyond the architectural way of thinking.*
**Student 2**	*it was hard to communicate with the students from the other disciplines because we were not standing on a common ground of knowledge. For example, we as architects had to make decisions regarding the electrical discipline because of the lack of communication with the ELEC Students.*
**Student 3**	*Other disciplines could not understand architecture work, which caused communication difficulties at the first stages of design. In contrary, we as architecture students could grasp other disciplines’ work in accurate.*
**Student 4**	*communication with the other disciplines was somehow useful. However, the more important was the deep research of the proper design systems and applying them to the project as models with taking into consideration the other disciplines’ systems to avoid clashes.*

**Fig. 3:**
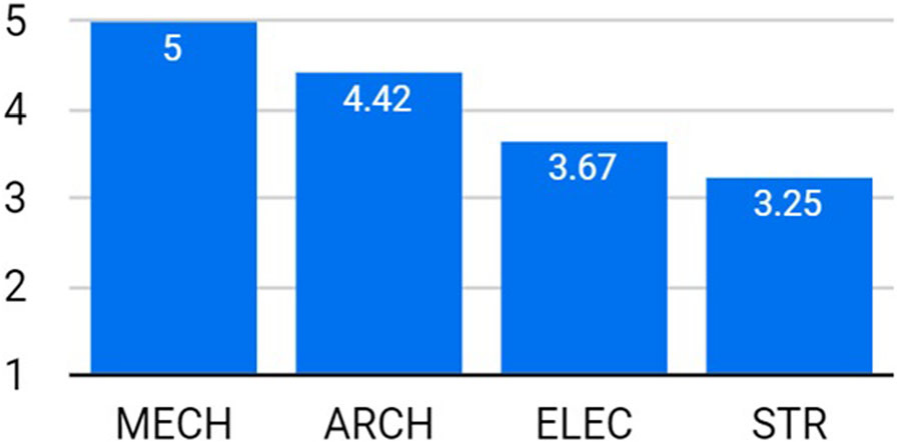
Level of understanding of the integrated design process for all disciplines

**Fig. 4:**
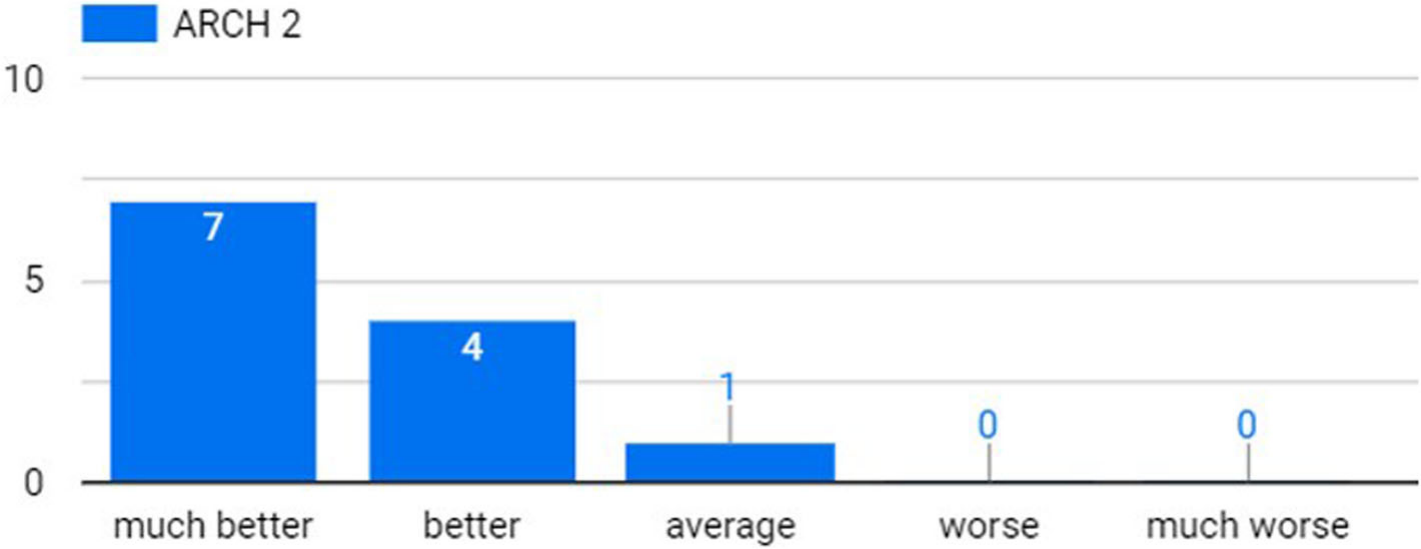
For ARCH students, level of understanding of the integrated design process in IDC, compared to the TDS

Figure 3 shows that all students had a satisfying understanding of the integrated design process. In addition, almost all ARCH students thought that such understanding would be attained by the IDC, much better than by the TDS (Fig. 4). Specifically, two students (9%) claimed that they have learned the process of integrated/actual design, which was not fully realized before the experiment, while five students (23%) were specifically indicating the rising of awareness of multi-discipline clash reconciliation. Three students (14%) mentioned that the lectures introduced by the instructors from different disciplines were helpful and useful in the design process.

Negative feedback regarding course schedule and course administration was received from most of the students. First, 11 students (50%) complained about the lack of adaptability to the exceptional circumstances, indicating the period of closure due to the COVID-19 pandemic. Six students (27%) stated that the administration should have had more strictness with the instructors, since the instructional following up was weak or, according to some students, was almost absent. Three students (14%) mentioned that this caused poor technical knowledge. Not only the instructors who were said to lack commitment, but three students (14%) also complained about the unseriousness of their teammates. In addition, complaints were reported regarding the graduation project (GP)’s effect on students’ performance, which was clearly stated by three students (14%).

On the other hand, there was positive feedback from seven students (32%) who showed their satisfaction with designing a semi-real project as it is considered a practical training under the academic supervision. In addition, some students commended that the experiment was being run as a competition, which gave them much encouragement.

**Fig. 5:**
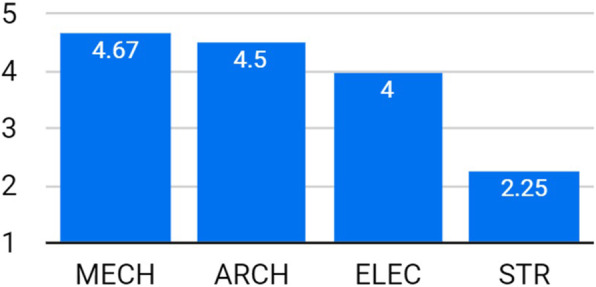
Level of understanding BIM for all disciplines

**Fig. 6:**
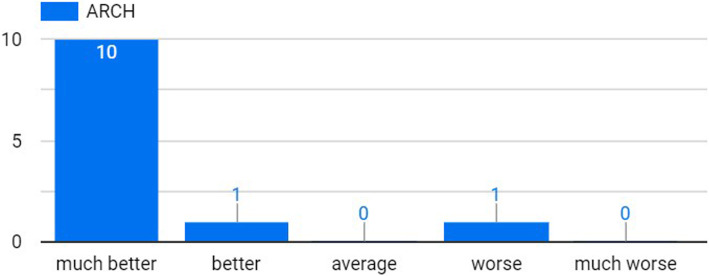
For ARCH students, level of understanding BIM in IDC, compared to the TDS

The students were asked if using BIM software helps in the collaborative design. Figure 5 shows that other than the STR students, positive evaluation was dominant. In addition, almost all ARCH students claimed that the IDC had very high benefits for them in mastering BIM software, compared to the TDS (Fig. 6). It is noticed from Figs. 3 and 5 that the STR students gained the lowest understanding compared to their colleagues. The reason, according to their comments, was the conflict in their academic schedules. They were devoting the majority of their time to GP and therefore were unable to fully engage in the IDC.

Eleven students (50%) have reported that they have gained more understanding of BIM technology or mastering Revit software through the experiment. For example, Student 4 claimed that the discussion with the colleagues from all disciplines had helped grasping how to deal with the software. He stated: “for example, in the clash detection task, the understanding the other disciplines’ delicate needs created specific problems and sub-tasks, which helped deep searching and finding the best software techniques to solve them.” However, all MECH and ELEC students (27% of all) stated that they faced barriers while using software to design. Thus, one can observe the significant variance in feedback between ARCH and students from other disciplines. This can be explained because of the previous experience of ARCH students in using such software.

The results of the experiment have shown that the hypothesis is likely true. ARCH students manifested a satisfying understanding of other disciplines’ knowledge as well as the integrated design process itself. In addition, they thought that such understanding would be attained by the IDC, much better than by the TDS. However, ARCH students played a dominant role more than expected. They were much more interactive with themselves, while less interactive with their colleagues from the other disciplines.

It is clearly noticed from the students’ feedback that providing prior knowledge required for the IDC is highly recommended. For example, they recommended early provision of BIM software tutorials before starting the experiment. In addition, students reported that the opportunity of applying the IDC should be expanded by mainly allowing more dedicated time. Specifically, they suggest (1) increasing meetings hours on campus; (2) reducing the size of the pilot project, in which less time is required in earlier stages of design; (3) applying the IDC to be a part of the GP, which would cause no busyness due to the GP; and (4) offering the IDC as a compulsory rather than an elective course.

Other suggestions were relevant to the improvement of the quality of interdisciplinary design. For example, some students preferred that only top students should be chosen. They also suggested that communication should be done with graduated students and professionals besides the instructors in order to gain more design experience.

## Conclusions

The study has clarified the shortcoming of the existing literature on AEC interdisciplinary design education. In response, it has offered an alternative model of interdisciplinary design studio employed as an elective course in architectural and other engineering departments. The study has adopted a qualitative research methodology to highlight the value of interdisciplinary design studio and to test the hypothesis. An experiment has been designed and then applied with a designed case study. Using observation, interviews, and surveys, data have been gathered and then analyzed to identify the experiment’s effectiveness, challenges, and students’ own experiences, in comparison with the traditional design studio.

The results of the experiment have shown that the hypothesis is satisfactory. All students expressed considerable satisfaction with the IDC. They claimed that the collaboration has raised the awareness of each other’s knowledge and the understanding of the integrated design process. However, they have reported negative feedback regarding the course schedule and course administration. ARCH students were better at delivering their knowledge than were other disciplines. They were more aware of other disciplines’ work and had more understanding of BIM and Revit software. In addition, the IDC has been proved by all ARCH students to be more valuable than the TDS in the understanding of the integrated design process and mastering BIM software.

The chosen programs that documented experiments of teaching interdisciplinary design are limited to the same country (USA), having similar university settings. Studying a variety of programs for universities from different environments and cultures would be more convincing. In addition, the introduced methods for the application of the IDC in the design studio are preliminary and suggested, carried out on a small sample. A bigger sample could offer more reliable results. Other researchers are encouraged to test the proposed framework and research methods further or to introduce other methods to highlight the value of interdisciplinary design studio in architecture or other engineering departments.

The paper fulfills the existing need to study how collaborative teaching can be adopted to teach AEC interdisciplinary design as an elective course in architectural and other related engineering departments, i.e., structural, electrical, and mechanical engineering departments. The paper’s contribution will have a wide-range impact. It can guide the educators in architectural and engineering programs, locally or internationally, in establishing new curricula and developing existing ones.

## Supplementary Information

**Additional file 1.** Post-experiment survey.

## Data Availability

Not applicable.

## Abbreviations

AEC: Architecture, Engineering, and Construction
ILO: Intended Learning Outcome
BIM: Building Information Modeling
IDC: Interdisciplinary Design Course
TDS: Traditional Design Studio
ARCH: Architectural Engineering
STR: Structural Engineering
ELEC: Electrical Power Engineering
MECH: Mechanical Power Engineering
GP: Graduation project
