# Supplementary figures and images for: Interdisciplinary design education: development of an elective course in architecture and engineering departments
Source: J. Eng. Appl. Sci. 2021 Sep 22;68(1):9. doi: 10.1186/s44147-021-00010-2 (PMC8455157; doi:10.1186/s44147-021-00010-2)

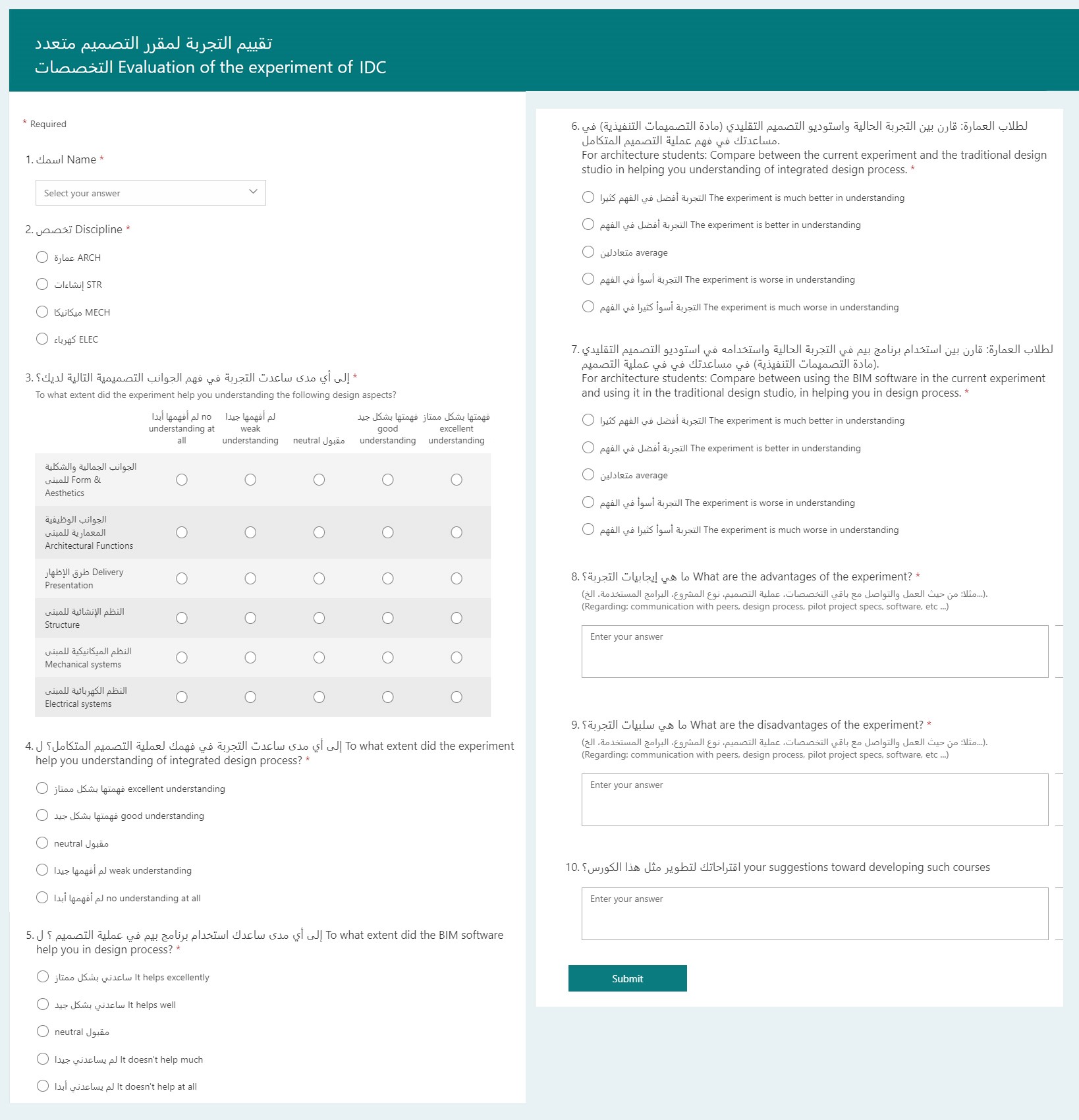

Supplement: Supplementary file 1 — Additional file 1. Post-experiment survey. [file 44147_2021_10_MOESM1_ESM.jpg]
